# A Comparative Study of the Attachment of the Teeth

**Published:** 1902-09

**Authors:** Frederick B. Noyes


### A COMPARATIVE STUDY OF THE ATTACHMENT OF THE TEETH.[1]

BY FREDERICK B. NOYES, B.A., D.D.S.

THAT the teeth are not a part of the osseous system, but are appendages of the skin, supported, in man, by a special development of bone forming the alveolar ridges of the maxillary bones, is as well established as any fact concerning human dentition. The work of Oscar Hertwig, "Ueber Bau und Entwickelung der Placoidschuppen," published in *Jenaische Zeitschrift,* 1874, established very clearly the homology existing between the teeth and the dermal or placoid scales of the ganoid, silurioid, and dipnoan fishes, both as to similarity of structure and development.

---

[1] Read before the Section on Stomatology of the American Medical Association, at Saratoga Springs, June, 1902.

Much has been written descriptive of the teeth of various animals, their modifications of form, and attachment to adapt them to modifications of function, and various classifications of the means of attachment have been made. Of these, perhaps the best and most logical is given by Charles Tomes in his "Dental Anatomy," describing four forms of attachment,—1, by fibrous membrane; 2, by hinge-joint; 3, by ankylosis; 4, by insertion in a socket.

I wish simply to take up these various forms of attachment, and show, if possible, the comparison between them, and the evolution of the more complicated forms from the simpler. We must begin with an examination of the structure and attachment of the placoid scales and the simplest form of tooth as illustrated in the shark.

The dermal scales are composed of a conical cap of calcified tissue developed from within outward, by an epithelial organ, and corresponding in structure to the enamel. This cap is supported upon a conical papilla of calcified tissue formed from without inward, and corresponding to dentine. In the outer layer the arrangement of the fine tubules through the calcified matrix corresponds very closely to human dentine, but in the inner portions it is to be understood only by considering the formation of the dentine as progressing irregularly over the surface of the pulp and so dividing the pulp-tissue into portions enclosed in large canals, from which the fine tubules radiate. The base of this partially calcified papilla has a calcified connective tissue built onto it by the derma, which corresponds to cementum forming the basal plate, spreading out more or less in the connective-tissue layer of the skin, and into which the fibres of this layer are built, so attaching the denticle or dermal scale to the deep layer of the coreum. This tissue very exactly resembles cementum. It is formed on the dentine as the cementum of a human tooth is, and shows the connective-tissue fibres embedded in it. In the ganoids the basal plates of adjoining scales unite, forming the armor plates of such fish as the sturgeon and gar-pike, and the dentical remains projecting from the surface of the plates.

In the simplest teeth, as of the shark lamna, which are typical dermal scales, we have an exactly similar method of attachment, which may be taken as the simplest and most rudimentary, or attachment in a fibrous membrane. That is, there is no development or modification of the arch of the jaw, and the teeth have no direct attachment to the bone; in fact, the jaws themselves are chiefly cartilage.

The formation of the hinge attachment as illustrated in many of the fishes may be understood as a modification of the attachment in a fibrous membrane in a more highly specialized creature. These hinged teeth are found in many fishes and in the poison-fangs of snakes. The jaws are calcified, and the basal plate or cementum may be considered as confined to, or specially developed on, one side of the dentine papilla, which is also more highly developed, especially in snakes. This cementum is built and calcified around the fibres of the fibrous tissue which pass directly to the bone of the jaw at that point. This bone is to be regarded as an addition to the jaw specially developed for each tooth. We have then not only a modification in the arrangement of the cementum, but a development of bone for attachment of the tooth. The blood-vessels pass through the fibres of the hinge to the pulp, and are not affected by the motion of the tooth on the hinge; in fact, the pulp seems to be attached to the hinge. There are many complications of this method of attachment, but this may be taken as the type and the manner of its modification from the rudimentary conditions. The distinction, in this form of attachment, from the dermal scale consists in a modification of the arrangement of the cementum of the basal plate and a development of bone from the jaw to attach fibres which pass from cementum to bone directly. It should also be said that there are developments in the hinge teeth related to the third form of attachment,—namely, ankylosis, which cannot be understood until this form is studied.

The third form of attachment, ankylosis, or direct calcified union with the bone of the jaw, cannot be understood without a careful study of the nature and formation of the dentine in these rudimentary teeth. It is evident, from a study of the dentine of the dermal scales, that, compared with human dentine, the tissue is rudimentary and not differentiated from other similar connective tissues. The tubules are comparatively very irregular, and resemble very strikingly the tubules found in the secondary dentine formed by a degenerating pulp. The odontoblasts, or dentine-forming cells, are not like the highly specialized cells which form the primary human dentine, but resemble very closely simple spindle-shaped connective-tissue cells; the nucleus is larger and oval in form, and the protoplasm stretches off from it in one direction into a fibril instead of in two directions into a spindle. The cells are much smaller than human odontoblasts and nearer the size of ordinary spindle-cells of the human pulp. In fact, they look more like specially developed spindle-cells than odontoblasts. The formation of dentine begins on the surface, at the apex of a cone-shaped papilla of connective tissue, and proceeds inward. If the formation continues uniformly over the surface of the papilla, a solid layer of fine tubuled dentine results, but it often proceeds irregularly, apparently having special reference to the neighborhood of blood-vessels, so that irregular projections of dentine are found on its inner surface, dividing the pulp more or less into portions inclosed in larger channels or tubes. These may be very regular in arrangement and form around blood-vessel loops embedding the blood-vessel in the calcified tissue, forming what has been called vaso- or vascular dentine; but the formation is still from the surface of the pulp until it is obliterated, except for what remains in the larger canals. As distinguished from this formation of dentine we find in the body of the dental papilla of many fishes the formation of spicules of calcified tissue, shooting down through the substance of the pulp which resemble neither dentine nor typical bone. They are more to be compared with the first formation of bone in membranes, or in the embryonal connective tissue of the body of the human jaw which is afterwards removed by absorption and replaced by true Haversian system bone. These calcifications contain lacunæ, and have tubules or canaliculi running through them, and so, as Tomes says, are intermediate between dentine and bone. They divide the pulp into irregular spaces, and interdigitate, or perhaps actually join, the formation of dentine which has been progressing from the surface of the pulp. These spicules run down into the bone of the jaw, forming an actual calcified attachment for the tooth with the jaw, but in this view of it it is to be regarded as a calcification, or rather a formation, of bone in the pulp-papilla interlocking with the dentine. In some of the fishes, as in Scarus, there is at the same time the remains of the cementum of the basal plate formed on the outside of the dentine around the base of the cone, which includes fibres which pass to the surface of the bone. Ankylosis is confined to the teeth of many fishes, and may be stated as a modification from the dermal scale, resulting in the reduction or loss of the basal plate and an ossification of the pulp continuing through the connective tissue at the base of the pulp to the body of the jaw.

The development of the fourth form of attachment, by implantation in a socket, seems to be an evolution starting from the same point but proceeding in a different direction. It is associated with the very great increase in the size of the teeth and consequent necessity for stronger attachment. This evolution is illustrated in the teeth of the reptiles. Wiedersheim classifies the teeth of reptiles as, 1, resting upon a ledge on the lingual side of the jaw, *pleurodont* dentition; 2, resting on the upper border of the jaw, with a slight ridge around them, *acrodont* dentition; 3, lodged in permanent alveoli, as in the crocodiles, *thecodont* dentition. These three classes illustrate three stages in the development of the socket method of attachment. In the simplest form there is a cone-shaped tooth attached to the bone around its base by fibres being built into the cementum and the bone. There is little modification of the rudimentary form and little development of bone for its attachment. In the higher form the tooth has become long or peg-shaped, and the bone has grown up around a portion of it to support it, but it is attached to the bone by connective-tissue fibres being built into the cementum on the surface of the peg and into the bone of attachment on the jaw. The development of the form of the tooth to the peg from the cone may be understood as a continuing of the development of odontoblasts and the formation of dentine (which always begins at the apex of the cone) farther and farther down on the sides of the dental papilla; then the formation of cementum which begins around the base of the cone and continues down on the outside of the calcified dentine, covering its outer surface and building the connective-tissue fibres into the tooth. The development of the bone accompanies, or rather follows, that of the tooth, building the other ends of these fibres into the bone which is developed to support the tooth.

While this probably represents, in a rough way, the manner of evolution and differentiation of the teeth and their attachment, as shown by the works already published in this field, there is still some work to be done in the study of the development and specialization of the dental tissues, and especially in the study of the relation between the bone of attachment and the cementum. I had hoped to have some things to present in this study, but the failure of material makes it impossible at this time.

